# Studies in a Murine Model Confirm the Safety of Griffithsin and Advocate Its Further Development as a Microbicide Targeting HIV-1 and Other Enveloped Viruses

**DOI:** 10.3390/v8110311

**Published:** 2016-11-17

**Authors:** Joseph Calvin Kouokam, Amanda B. Lasnik, Kenneth E. Palmer

**Affiliations:** 1 Department of Pharmacology and Toxicology, University of Louisville School of Medicine, University of Louisville, Louisville, KY 40202, USA; 2 James Graham Brown Cancer Center, University of Louisville School of Medicine, University of Louisville, Louisville, KY 40202, USA; amanda.lasnik@louisville.edu; 3 Center for Predictive Medicine, University of Louisville, Louisville, KY 40202, USA

**Keywords:** Griffithsin, HIV, safety, mouse model, microbicide, peripheral blood mononuclear cells (PBMCs)

## Abstract

Griffithsin (GRFT), a lectin from *Griffithsia* species, inhibits human immunodeficiency virus-1 (HIV-1) replication at sub-nanomolar concentrations, with limited cellular toxicity. However, in vivo safety of GRFT is not fully understood, especially following parenteral administration. We first assessed GRFT’s effects in vitro, on mouse peripheral blood mononuclear cell (mPBMC) viability, mitogenicity, and activation using flow-cytometry, as well as cytokine secretion through enzyme-linked immunosorbent assay (ELISA). Toxicological properties of GRFT were determined after a single subcutaneous administration of 50 mg/kg or 14 daily doses of 10 mg/kg in BALB/c mice. In the context of microbicide development, toxicity of GRFT at 2 mg/kg was determined after subcutaneous, intravaginal, and intraperitoneal administrations, respectively. Interestingly, GRFT caused no significant cell death, mitogenicity, activation, or cytokine release in mPBMCs, validating the usefulness of a mouse model. An excellent safety profile for GRFT was obtained in vivo: no overt changes were observed in animal fitness, blood chemistry or CBC parameters. Following GRFT treatment, reversible splenomegaly was observed with activation of certain spleen B and T cells. However, spleen tissues were not pathologically altered by GRFT (either with a single high dose or chronic doses). Finally, no detectable toxicity was found after mucosal or systemic treatment with 2 mg/kg GRFT, which should be further developed as a microbicide for HIV prevention.

## 1. Introduction

Despite recent advances in the development of new antiretrovirals, human immunodeficiency virus (HIV) continues to spread worldwide with an astonishing rate of approximately 5500 new infections a day [1]. HIV is a highly mutable virus and, thus, a major limitation of existing antiretrovirals is the ability of HIV to develop resistance under selective pressure from these drugs. This has led drug designers to opt for complementary combinations with high manufacturing costs, which makes delivery in resource-poor areas (where the disease burden is often highest) unfeasible [2]. Therefore, new, more efficient, and inexpensive antiviral drugs are needed in order to better combat HIV/AIDS. A few years ago, an anti-HIV bioassay guided fractionation of aqueous extracts of the red alga *Griffithsia* species collected from the waters off New Zealand lead to the discovery of a lectin termed Griffithsin (GRFT) [3]. Thanks to its pronounced anti-viral activity against both laboratory and primary isolates of HIV in the picomolar range, GRFT is considered a potential candidate microbicide to prevent the sexual transmission of HIV and AIDS [3,4,5].

Binding of oligosaccharides to multiple sites on a single molecule of GRFT provides the basis for its potent antiviral properties [6], and it has been hypothesized that the lectin would inactivate other enveloped viruses, especially those with highly glycosylated proteins on their surface. Indeed, GRFT was recently shown to tightly bind at multiple sites to the spike glycoprotein found on the envelope of the coronavirus (CoV), the etiologic agent of the severe acute respiratory syndrome (SARS) [7]. In the same work, a potent activity was reported for GRFT against a broad spectrum of coronaviruses in addition to its outstanding in vivo efficacy in SARS-CoV-infected mice [7]. More recently, GRFT was shown to inhibit Japanese encephalitis virus infection, both in vitro and in a mouse model of infection [8]. In addition, GRFT exhibits significant inhibitory activities on herpes simplex virus (HSV)-2, both in vitro and in a mouse model of genital herpes, and protects mice that harbor human primary hepatocytes in their liver from hepatitis C virus (HCV) infection [9,10,11]. GRFT is currently under development as a topical microbicide in our laboratory and others in order to prevent the sexual transmission of HIV. The outcomes of recent clinical trials have highlighted the shortcomings of current preclinical safety assays for microbicide product development [12,13,14,15]. Interestingly, several studies have revealed that GRFT has an excellent safety profile in vitro, ex vivo using cervical explants, and in the rabbit vaginal irritation model, considered the gold standard of preclinical safety test for vaginal products [3,4,12,16]. Topical administration of GRFT is unlikely to result in the drug being present in the circulation. However, it is important to study systemic toxicity of microbicides in case of absorption after topical administration e.g., in patients with ulcerative sexually transmitted diseases (STDs). It is also important to evaluate the systemic toxicity of GRFT should this lectin be used for the treatment of blood borne pathogenic enveloped viruses, including HCV, hepatitis B (HBV), Ebola, Marburg, and Japanese encephalitis viruses. Importantly, we recently found that GRFT displays minimal toxicity in guinea pigs after chronic administration of 10 mg/kg daily subcutaneous doses [17].

In the present study, we first sought to validate the use of a murine model in safety studies by assessing the toxicological effects of GRFT on isolated mouse peripheral blood mononuclear cells (mPBMCs) in vitro. Our findings presented here corroborated our previous work using human PBMCs [12]. Then, we evaluated the toxicological properties of GRFT after parenteral administration of 50 and 10 mg/kg, respectively, of the drug in BALB/c mice. Only minimal toxicity was observed, even at these high doses. Finally, the effects of 2 mg/kg GRFT after intravaginal, intraperitoneal, and subcutaneous administrations were evaluated. The latter dose reflects the amount of GRFT that would be mucosally administered in the context of microbicide application as demonstrated in HSV-2 efficacy studies [9]. Interestingly, no toxicities were observed in these experiments, indicating an excellent in vivo safety profile for GRFT in the murine model.

Overall, GRFT’s safety profile confirms the lectin as an outstanding microbicide candidate which could become a powerful weapon in the fight against human immunodeficiency virus-1 (HIV-1) and other blood-borne enveloped viruses.

## 2. Materials and Methods

### 2.1. Griffithsin (GRFT) and Other Lectin Reagents

Griffithsin (GRFT) is a 12.7 kDa carbohydrate binding protein that was first isolated from the red alga *Griffithsia* species. The recombinant GRFT used in these studies was produced in *Nicotiana benthamiana*, a close relative of tobacco, according to the procedures described for a scalable manufacture of the lectin [16]. The control lectins Concanavalin A (ConA) and phytohemagglutinin (PHA) A were purchased from Sigma Aldrich (St. Louis, MO, USA).

### 2.2. In Vitro Effects of GRFT in Mouse Peripheral Blood Mononuclear Cell (mPBMCs)

#### 2.2.1. Mouse PBMC Isolation and Culture

Mouse PBMCs were isolated from adult female untreated BALB/c mice (The Jackson Laboratory; Bar Harbor, ME, USA) using a Ficoll gradient centrifugation. Briefly, sterile blood samples were collected in heparin-coated VACUTAINERs^®^ (BD, Franklin Lakes, NJ, USA) and transported within one hour to our laboratory at ambient temperature. Blood samples were diluted about two times with Hanks balanced salt solution (HBSS) and the resulting solution was carefully added to an overlay of 10 mL LMS^®^-Lymphocyte separation medium (Ficoll and sodium diatrizoate) manufactured by MP Biomedicals (Irvine, CA, USA) in a 50 mL conical tube, creating a sharp blood- Lymphocyte Separation Medium (LSM) interphase. After low speed centrifugation (400× *g*, 30 min) with no brake, mPBMCs were collected at the interface layer between plasma and Ficoll. The cells were then washed twice with HBSS and counted for recovery and viability using 0.4% Trypan Blue (Invitrogen Corporation, Grand Island, NY, USA). Mouse PBMCs were cultured in RPMI-1640 supplemented with 10% fetal bovine serum (FBS) and an antibiotic cocktail containing penicillin and streptomycin in a humid environment with 5% CO_2_ at 37 °C.

#### 2.2.2. Cytokine Release

To evaluate the effect of GRFT on cytokine release in mPBMCs, cells were treated for 24, 48, and 72 h with 1 or 4 μM GRFT, with culture supernatants collected at each time point. Interleukin (IL) 1 beta (1b), IL 6 (IL-6), IL 10 (IL-10), and tumor necrosis factor alpha (TNF-α) amounts in supernatants were assessed by enzyme linked immunosorbent assay (ELISA) using specific ELISA Ready-SET-Go! kits from eBioscience (San Diego, CA, USA), following the manufacturer’s instructions. In these experiments, ConA (0.37 and 1 μM) and phosphate-buffered saline (PBS) were used as positive and negative controls, respectively, as previously described [12].

#### 2.2.3. Cell Viability and Mitogenicity

For cell viability and mitogenicity experiments, cells were cultured for 72 h in presence of GRFT (1 and 4 μM) or controls and analyzed flow-cytometrically for changes in size and/or granularity (mitogenicity) or propidium iodide (PI) uptake (cell death).

#### 2.2.4. Surface Activation Marker Levels

To assess the expression of cellular surface activation markers, flow cytometry was performed after dual fluorescent staining with anti-mouse antibodies purchased from BD Biosciences^TM^ (San Diego, CA, USA). Briefly, cultures were transferred from plates to 5 mL round-bottom tubes, and washed with PBS containing 5% inactivated FBS. Then, cells were blocked with purified rat anti-mouse CD16/CD32 (Mouse BD Fc Block^TM^; BD, San Jose, CA, USA) for 10 min followed by incubation in the dark with fluorescein isothiocyanate (FITC)-conjugated rat anti-CD4 mAb in combination with phycoerythrin (PE)-conjugated rat anti-CD25 or hamster anti-CD69 mAb for 30 min on ice. Finally, cells were washed and analyzed with a FACSCalibur (BD, San Jose, CA, USA). All flow cytometry data were acquired and analyzed using CellQuest Pro from BD, counting 10,000 events per sample. ConA (0.37 μM) and PHA (10 μg/mL) were used as positive controls [12,18], and PBS as the negative control.

### 2.3. In Vivo Effects of GRFT in a Mouse Model

#### 2.3.1. Animal Housing

Six to eight week-old female BALB/c mice were purchased from The Jackson Laboratory (JAX), Bar Harbor, ME, USA. The animals were housed in filtertop microisolator cages in a temperature- and humidity-controlled room with an alternating light/dark cycle of 12 h, with standard mouse diet and water ad libitum. All experimental procedures were approved by the University of Louisville’s Institutional Animal Care and Use Committee. Mice were allowed approximately one week of adaptation before treatment.

#### 2.3.2. Subcutaneous (s.c.) Injections and Sample Collection

In the first experiment, groups of four animals were injected with either 10 mg/kg dose of GRFT in 100 μL PBS or the vehicle only at Day 1. Animal survival, behavior, and fitness were recorded after one and two weeks, and animals were euthanized at Day 15. In parallel, a higher single dose of 50 mg/kg GRFT was administered to 30 mice divided into three subgroups of 10 evaluated at one, seven, and 14 days post-treatment, respectively. As controls, three groups of five animals were treated with the vehicle PBS. Next, we studied the effects of GRFT after chronic administration. Here, two groups of 15 mice each were treated with 10 mg/kg GRFT or PBS on a daily basis for 14 days. Animals were sacrificed at Day 14 (nine mice per treatment group), Days 16 and 21 (three per treatment group at each time point, respectively). In all experiments, blood was collected immediately after euthanasia, and blood samples were submitted to complete blood count (CBC). In addition, plasma samples were evaluated for a panel of markers of organ toxicity, using blood chemistry techniques. After euthanasia, spleens, lungs, kidneys, livers, and hearts were extracted and weighed. In the case of the chronic treatment, kidney, liver, and spleen tissues were stained for histopathology studies. In addition, spleen cells were isolated and assessed for activation. In the last experiment, mice were injected with 10 daily doses of 2 mg/kg GRFT in 100 µL PBS, or vehicle only. Toxicity was assessed by measuring body and spleen weights, as well as splenocyte activation.

#### 2.3.3. Intravaginal and Intraperitoneal Administrations, and Sample Collection

Eight to 10 weeks old mice were administered 10 daily vaginal applications of 0.1% GRFT formulated in 40 µL Carbopol 974P, glycerin, ethylenediaminetetraacetic acid (EDTA), methyl-paraben, and propyl-paraben as previously described [9]. A matched Carbopol placebo gel containing all excipients but no GRFT was used as a negative control. For intraperitoneal treatment, mice were injected with 2 mg/kg GRFT in 100 µL of PBS or the same volume of vehicle for 10 days. Toxicity in both cases was assessed by measuring body and spleen weights as well as splenocyte activation.

#### 2.3.4. Splenocyte Activation

Spleen cells were isolated according to a well-established procedure [19] using ammonium-chloride-potassium (ACK) lysis buffer to remove red blood cells. Spleen T-cell activation was studied by flow cytometry as described above with mouse PBMCs for the detection of CD4, CD25, and CD69. B-cell activation was studied in a similar fashion using a dual fluorescent staining with anti-mouse CD19 (FITC-conjugated) and B220 (PE-conjugated) antibodies purchased from BD Pharmingen (San Jose, CA, USA).

#### 2.3.5. Histopathology

Kidney, spleen, and liver tissues were collected from animals treated with 14 daily doses of 10 mg/kg of GRFT, on the last treatment day (Day 14) or after a week recovery (Day 21) and fixed in 10% neutral buffered formalin. Paraffin-embedded sections were stained with hematoxylin and eosin (H and E staining) and evaluated in a blinded manner by a trained pathologist.

#### 2.3.6. Hematology Parameters

Complete blood count was carried out on a HemaVet 950FS Hematology Analyzer (Drew Scientific group, Inc., Dallas, TX, USA) standardized for mouse blood. This instrument uses flow cytometric techniques to generate the following parameters in potassium-EDTA anticoagulated whole blood: red blood cells (RBC, 10^4^/µL), total and differential leukocyte count (neutrophils, lymphocytes, monocytes, eosinophils, and basophils as 10^3^/µL or %), hemoglobin concentration (HGB, g/dL), hematocrit (HCT, %), mean corpuscular volume (MCV, fl), mean cell hemoglobin (MCH, pg), mean cell hemoglobin concentration (MCHC, g/dL), red cell distribution width (RDW, %), platelets (PLT, 10^4^/µL), and mean platelet volume (MPV, fl).

#### 2.3.7. Blood Chemistry

Biochemical analyses were performed using a VetTest 8008 Chemistry Analyzer (IDEXX Laboratories, Inc., Atlanta, GA, USA). The collected blood samples were used to assess the plasma levels of total protein (TP), globulin (GLOB), albumin (Alb), blood urea nitrogen (BUN), creatinine (CREAT), total cholesterol (Chol), aspartate-aminotransferase (AST), alanine-aminotransferase (ALT), alkaline phosphatase (ALP), amylase (AMS), total bilirubin (TBILI), blood glucose (BG), sodium (Na), potassium (K), calcium (Ca), and phosphorus (P), using reagents and methods provided by IDEXX Laboratories, Inc., Atlanta, GA, USA.

### 2.4. Statistical Analysis

Group means and standard deviations were derived from body- and organ-weights, hematology values, blood chemistry parameters, cytokine concentrations in cell supernatants, and cells in a defined region of the flow-cytograms. Statistical significance was assessed by a one-way analysis of variance (ANOVA) and Student’s *t*-test unless otherwise stated, using GraphPad software (San Diego, CA, USA). Differences were considered statistically significant if *p* < 0.05.

## 3. Results

### 3.1. In Vitro Effects of GRFT on Purified mPBMCs

**Figure 1:**
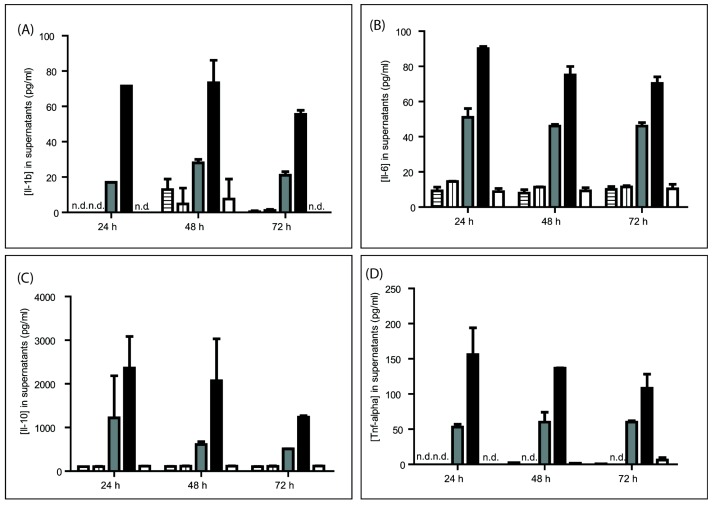
Effect of Griffithsin (GRFT) on the secretion of some mediators of the immune response. Supernatants were collected from mouse peripheral blood mononuclear cell (mPBMC) cultures treated with 1 and 4 μM GRFT (horizontal and vertical lines, respectively), 0.37 and 1 μM Concanavalin A (ConA; grey and black, respectively) and phosphate-buffered saline (PBS; white) for 24, 48, and 72 h. Interleukin (IL) 1 beta (1b) (**A**); IL 6 (**B**); IL 10 (**C**); and tumor necrosis factor alpha (TNF-α) (**D**) amounts in the supernatants were assessed by enzyme-linked immunosorbent assay (ELISA).

First, we evaluated the levels of four key cytokines, including IL-1b, IL-6, IL-10, and TNF-α, in mPBMC culture supernatants after 24, 48, and 72 h of incubation. GRFT at concentrations of up to 4 μM did not affect IL-1b and IL-10 release in mouse cells (Figure 1A,C). In contrast, ConA induced a massive cytokine secretion of all tested molecules in a concentration dependent manner (Figure 1). In the case of IL-6, there was no change in cytokine release after treatment with 1 μM GRFT (Figure 1B). However a slight, but non-significant, increase was observed after treatment with 4 μM GRFT, and this initial and transient effect was not noticeable by 48 h (Figure 1B). TNF-α concentrations were elevated after treatment with ConA but not with GRFT, where most values fell below detection levels, similar to PBS treated cells (Figure 1D). These data indicate that GRFT does not cause the release of the above key cytokines and chemokines assessed in mouse PBMCs.

**Figure 2:**
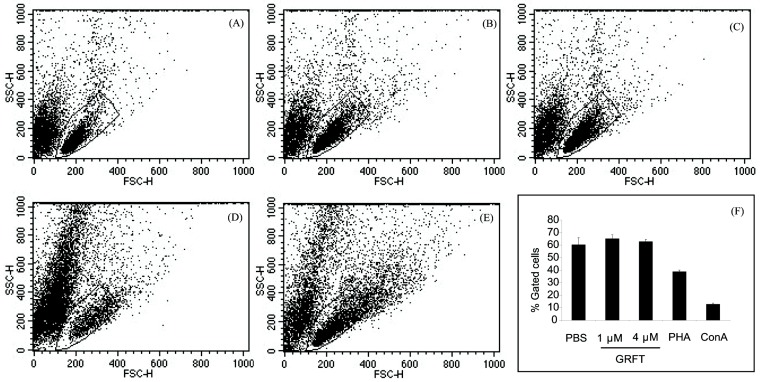
Mitogenic properties of GRFT on mPBMCs. Cells were cultured for three days in the presence of PBS (**A**); 1 and 4 μM GRFT ((**B**,**C**), respectively); 0.37 μM ConA (**D**) and 10 μg/mL phytohemagglutinin (PHA) A (**E**). Dot plots reveal forward scatter (FSC) and side scatter (SSC) of cells after treatment. Typical inactivated mPBMC subpopulations were gated and quantitated (**F**).

Next, we assessed the effect of GRFT on mPBMC size and granularity. When cultured in the presence of 1 and 4 µM GRFT, cells showed no difference by flow cytometry analysis compared to PBS treated cells, whereas cells treated with ConA and PHA showed sub-populations of cells with higher forward scatter (FSC) and/or increased side scatter (SSC) values, likely representing activated and enlarged PBMCs. This resulted in a reduction of typical PBMC number as gated in Figure 2A–E and quantified in Figure 2F. These observations were made even without the use of any fluorophore. Likewise, when the cells were loaded with propidium iodide (PI) which discriminates live from dead cells, flow cytometry histograms of mPBMCs treated with GRFT (1 or 4 µM) were similar to that obtained for PBS treated cells (Figure S1). PHA and ConA induced a pronounced cytotoxicity reflected by a shift in histograms (increased PI staining) in comparison with PBS- or GRFT-treated cells (Figure S1).

**Figure 3:**
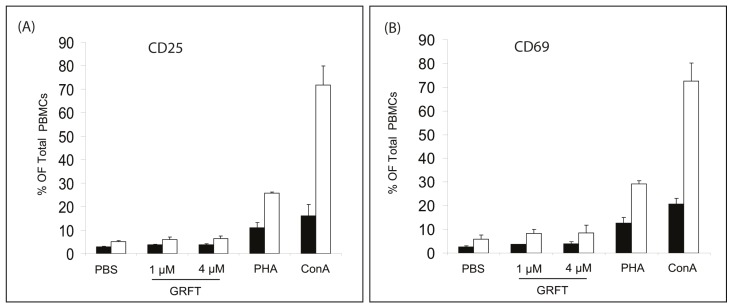
Effect of GRFT on the expression of cell surface activation markers in mPBMCs. Flow cytometry was performed after dual fluorescent staining with fluorescein isothiocyanate (FITC)-conjugated rat anti-CD4 mAb in combination with phycoerythrin (PE)-conjugated rat anti-CD25 or hamster anti-CD69. Cells were incubated in presence of PBS, 1 and 4 μM GRFT 0.37 μM ConA and 10 μg/mL PHA as indicated. (**A**) Represents the percentages of CD4+/CD25+ (black columns) and total CD25+ (white columns), whereas CD4+/CD69+ (black columns) and total CD69+ (white columns) ratios were plotted in (**B**).

Furthermore, we evaluated the percentages of cells expressing two known PBMC cellular activation markers, CD25 and CD69. In PBS-treated cells, 3.0% ± 0.2% of cells were CD4+CD25+ (Figure 3A). mPBMCs incubated in the presence of 1 and 4 μM GRFT showed similar values for CD4+/CD25+ cells with 3.7% ± 0.23% and 3.8% ± 0.4%, respectively, whereas these amounts were markedly increased by 87 nM, i.e., 10 µg/mL PHA (11.0% ± 2.3%) and 0.37 μM ConA (16.7% ± 4.8%), as shown in Figure 3A. When total numbers of cells expressing CD25 were compared, there was no statistically significant difference between PBS-treated cells (5.0% ± 0.5%) and those cultured in the presence of GRFT, 1 μM (5.9% ± 1.1%) or 4 μM (6.5% ± 1.0%) (Figure 3A). In contrast, 25.8% ± 0.3% and 71.7% ± 8.1% cells expressed CD25 after treatment with PHA and ConA, respectively (Figure 3A). Similar observations were made for the surface activation marker CD69. As shown in Figure 3B, treatment with 1 and 4 μM GRFT resulted in 3.7% ± 0.1% and 3.9% ± 1.0% CD4+CD69+ PBMCs, respectively, values not significantly different from that obtained for PBS-treated cells (2.7% ± 0.4%). However, a considerable increase in the CD4+CD69+ population of mouse PBMCs was observed after treatment with 10 μg/mL PHA (12.7% ± 2.2%) and 0.37 μM ConA (20.8% ± 2.4%). In addition, the total percentage of CD69+ cells was similar in unstimulated PBS treated cells (6.0% ± 1.6%), 1 μM and 4 μM GRFT (8.3% ± 1.7% and 8.6% ± 3.2%, respectively) as shown in Figure 3B. mPBMCs stimulated with 10 μg/mL PHA resulted in 30% CD69+ cells whereas 0.37 μM ConA treatment yielded about 70% cells expressing CD69 (Figure 3B). Taken together, these findings indicated a good safety profile in mPBMCs.

### 3.2. GRFT Toxicity in Mice after Parenteral Administration of a Single Dose

**Figure 4:**
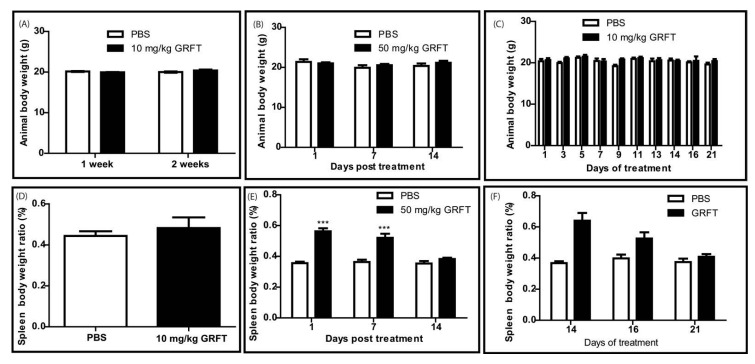
Evaluation of animal fitness and organ toxicity after subcutaneous (s.c.) administration of GRFT in mice. Mice were injected subcutaneously with a single dose of 10 mg/kg (**A**,**D**); 50 mg/kg (**B**,**E**), or 14 daily doses of 10 mg/kg GRFT (**C**,**F**). Animal body weights (**A**–**C**) and spleen weights (**D**–**F**) were recorded at the indicated time points (***, *p* < 0.001).

In a preliminary study, mice were injected with a single subcutaneous (s.c.) dose of 10 mg/kg GRFT. All animals survived the treatment after the two week observation period and their behavior (movement, activity, and grooming) was similar to that of mice treated with the PBS vehicle. Animal fitness was evaluated using body weight as a surrogate marker. One and two weeks after treatment, the GRFT group showed comparable body weights to control animals (Figure 4A). Organ toxicity was assessed in these studies by measuring organ weights at sacrifice. Hearts, lungs, livers, and kidneys were not affected by GRFT administration (data not shown). Spleen-to-body weight ratios were slightly elevated two weeks after treatment with 10 mg/kg GRFT (0.48% ± 0.10% vs. 0.44% ± 0.04% for PBS), but the difference was not significant (Figure 4D).

Since we did not observe any harmful effects after a single dose of 10 mg/kg in vivo, we were interested in evaluating the potential toxicity of single s.c. injection of a higher dose of 50 mg/kg GRFT in mice. The survival rate in this experiment was 100%, and the animals treated with 50 mg/kg GRFT did not show any changes in behavior or body weight (Figure 4B). These findings suggested that single doses of GRFT at 50 mg/kg did not cause massive toxicity when injected into mice. Mice were then euthanized one, seven, and 14 days post treatment. At all time-points, heart, lung, liver, and kidney weights were unaffected by the presence of GRFT (data not shown). However, spleen size and weight were increased for the animals sacrificed one day post treatment with a mean spleen body weight ratio of 0.56% ± 0.06% for GRFT-treated mice vs. 0.36% ± 0.02% for the PBS group (Figure 4E). Although decreasing, the splenomegaly persisted for one week after treatment with ratios of 0.52% ± 0.08% and 0.36% ± 0.03% for GRFT and PBS groups, respectively (Figure 4E). Interestingly, GRFT-treated animals had spleens comparable in size and weight to those of the PBS group after two weeks of recovery (ratios of 0.38% ± 0.03% for GRFT animals and 0.35% ± 0.03% for PBS controls) as shown in Figure 4F. Blood chemistry parameters in mice revealed no significant treatment-related differences between GRFT and control groups.

Likewise, the functions of kidney (BUN and CRE), pancreas (AMY, GLU), immune system (GLOB), and most indicators of liver function (ALB, TBIL, and ALT) were not adversely affected by GRFT treatment (Table S1). However, a non-significant increase was observed in ALP levels in samples collected one week after GRFT treatment (147.3 ± 7.8 arbitrary units (AU) vs. 100.0 ± 33.1 AU for PBS controls, *p* = 0.07). Two weeks after administration the same trend was observed with 144.2 ± 49.3 AU recorded in the GRFT group and 100.0 ± 5.3 AU for PBS treated animals (*p* = 0.1933). Furthermore, blood chemistry profiles revealed no effect of GRFT on the nutritional status (TP), calcium (Ca), phosphorous (PHOS), and plasma sodium (Na) (Table S1). However, a non-significant increase was noticeable in cholesterol amounts, with 107.0 ± 48.6 mg/dL in GRFT-treated animals compared with 63.3 ± 9.1 mg/dL found in the control group (*p* = 0.1817) after two weeks of recovery (Table S1). Blood samples were submitted to complete blood count (CBC) and there were no significant variations in the hematological profile due to GRFT treatment. Indeed, the values obtained after CBC for white blood cells (total leucocytes, NE, LY, MO, EO, and BA), erythrocytes (total count, HGB, HCT, MCV, MCH, MCHC, and RDW), and platelets (total count and mean platelet volume) were similar in both GRFT- and PBS-treated groups (Table S2).

### 3.3. GRFT Toxicity in Mice after Administration of a Chronic Dose of 10 mg/kg

**Figure 5:**
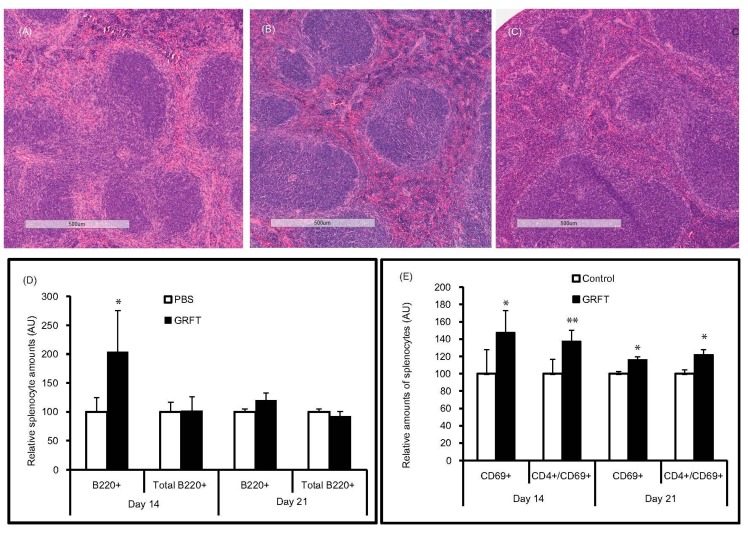
Effect of chronic GRFT treatment on spleen tissue and cell activation. (**A**–**C**) hematoxylin and eosin (H and E) stained spleens showing no, to minor, changes in spleen histology after 14 daily treatments with GRFT at 10 mg/kg. (**A**,**B**) are representative spleen specimens from the GRFT group at treatment end (Day 14) and Day 21 (14 days of treatment followed by a week of recovery), respectively; (**C**) representative spleen specimen from the PBS group at Day 14; flow cytometry was carried out after dual fluorescent staining with FITC-conjugated anti-CD4 or CD19 mAb in combination with PE-conjugated anti-B220 (**D**) or anti-CD69 (**E**). Relative amounts of splenocytes expressing a given surface marker were obtained with the PBS group value set at 100. B220+, cells staining positive for B220 only (single positive); total B220+ represent B220 single positive and B220/CD19 double positive (*, *p* < 0.05; **, *p* < 0.01)) .

Fourteen daily doses of 10 mg/kg GRFT were injected s.c. to mice with a control group receiving the PBS vehicle only. All mice survived this treatment and animal behavior was similar in both GRFT and PBS treatment groups. Throughout the experiment, fitness (as determined by the animal body weight) was unchanged after treatment with GRFT (Figure 4C). In addition, there was no change in lung, kidney, liver, and heart weights after GRFT treatment (data not shown). However, splenomegaly was observed after sacrifice at Day 14: spleen/body weight ratios of 0.64% ± 0.15% and 0.37% ± 0.04% for GRFT and control groups, respectively (Figure 4F). Interestingly, splenomegaly was decreased considerably after two days of recovery, with spleen-to-body weight ratios of 0.52% ± 0.07% for animals treated with GRFT (vs. 0.4% ± 0.04% in the PBS group, *p* = 0.0546). Seven days post treatment, the difference in spleen/body ratios was not significant (0.41% ± 0.03% and 0.37% ± 0.04% for GRFT and PBS groups, respectively, *p* = 0.2874) as shown in Figure 4F. Of note, histological studies of kidneys and livers collected at Days 14 and 21 revealed no observable pathology induced by GRFT (data not shown). Despite the splenomegaly, we observed no adverse pathology due to GRFT treatment in spleen tissues after H and E staining. Representative spleens are shown in Figure 5A–C. Interestingly, segmental marginal zone lymphoid depletions were occasionally observed across all groups regardless of treatment. In order to study whether splenomegaly was due to spleen B- and T-lymphocyte activation, mice were treated for 14 days with 10 mg/kg GRFT. Relative to PBS-treated animals, we found that B220-only positive splenocytes were markedly increased in the GRFT group at Day 10 (210.21% ± 73.68% relative to PBS cells, *p* = 0.0151), a difference that vanished after a week of recovery (Figure 5D). Interestingly, there was no difference between relative amounts of all cells staining positive for B220 (CD19+/B220+ and B220+ only) in splenocytes obtained from PBS or GRFT animals at either time point (Figure 5D). As shown in Figure 5E, cells expressing the T-cell activation marker CD69 were slightly more abundant in animals treated with GRFT (148.10% ± 25.41% relative to the PBS group, *p* = 0.0221) at Day 10. By Day 17, the difference was markedly reduced with 116.24% ± 3.03% relative to PBS treated mice (*p* = 0.0295). Likewise, spleen cells expressing both CD4 and CD69 were slightly increased after 14 daily treatments with GRFT (137.28% ± 12.80% relative to PBS, *p* = 0.0043); this difference was reduced during recovery (121.95% ± 5.58% CD4+/CD69+ relative to PBS, *p* = 0.0488).

Next, we conducted blood chemistry studies and observed no difference due to chronic treatment with GRFT in nearly all parameters, including Alb, BUN, CREAT, TBILI, ALT, AMS, BG, GLOB, TP, Ca, P and Na (Table 1). As observed with the single dose of 50 mg/kg GRFT, Chol levels were elevated, reaching 118.75 ± 10.53 mg/dL at Day 14 (control PBS = 87.25 ± 5.37 mg/dL, *p* = 0.0018); after a week of recovery there was a sharp decrease of cholesterol levels in GRFT-treated animals, although the difference was still statistically significant compared with the control group (Table 1). In addition, ALP levels were increased as a consequence of GRFT treatment (159.9% ± 31.0% relative to the PBS treatment group set to 100%) as evaluated at Day 14; at Day 21, there was a slight decrease, but these levels remained higher in the GRFT group (Table 1).

CBC data revealed only minimal treatment-related differences between the GRFT and control groups. The values obtained for red blood cells (total count, hemoglobin concentration, hematocrit, mean corpuscular volume, mean cell hemoglobin, mean cell hemoglobin concentration, and red cell distribution width) and platelets (total count and mean platelet volume) were similar in both treatment groups at all time-points (Table 2). There was no noticeable difference in the total leukocyte count due to GRFT treatment. However, we observed an increase in neutrophil numbers at Day 14 in the GRFT group (2.7 ± 0.2 vs. 1.7 ± 0.6 in the PBS control group). Nevertheless, this difference was not statistically significant as assessed by a two-tailed unpaired *t*-test with Welch’s correction. Meanwhile, a decrease in lymphocyte numbers was noticed in the GRFT group (3.6 ± 0.4 vs. 5.4 ± 0.7 in controls) but both values fell within the expected range described for mice. As shown in Table 2, MO, EO, and BA numbers were unchanged. Notably, the hematological profiles of animals seven days post-treatment were similar in both GRFT and PBS groups. Taken together, these findings indicated a good safety profile for GRFT in the murine model after parenteral administration.

### 3.4. GRFT Toxicity in Mice after Administration of a Chronic Dose of 2 mg/kg

**Figure 6:**
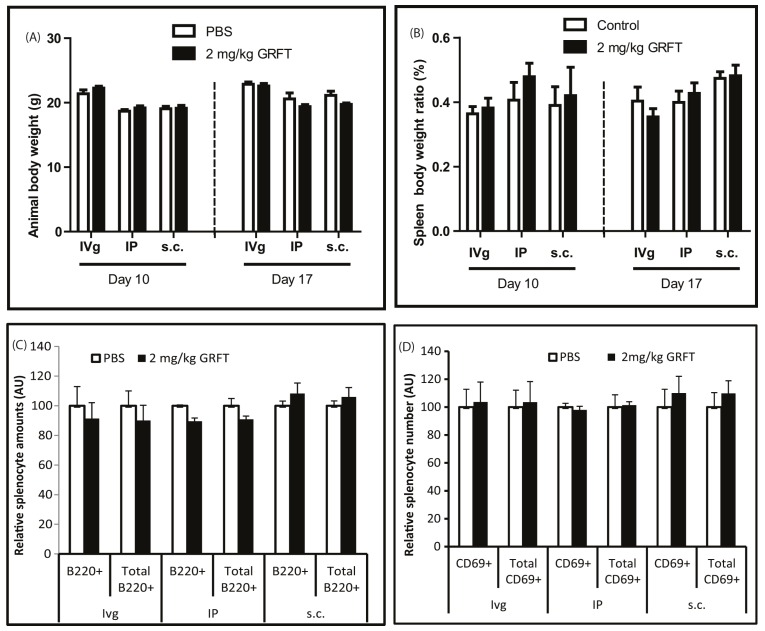
Safety of a chronic dose of 2 mg/kg GRFT in mice. Mouse body (**A**) and spleen (**B**) weights were assessed after 10 daily s.c., intravaginal, and intraperitoneal administrations, respectively, of GRFT at 2 mg/kg or PBS; In addition, immune cell activation of splenocytes from these mice was evaluated by flow cytometry after dual fluorescent staining with FITC-conjugated anti-CD4 or CD19 mAb in combination, respectively, with PE-conjugated anti-B220 (**C**) or anti-CD69 (**D**). B220+, cells staining positive for B220 only (single positive); Ttotal B220+ represent B220 single positive and B220/CD19 double positive.

Previous studies have shown that an intravaginal dose of 2 mg/kg GRFT is able to inhibit HSV-2, and we anticipate a similar dose to be used in the case of mucosal prevention of HIV-1. Therefore, we evaluated the toxicity of 2 mg/kg GRFT after intravaginal administration. We were also interested in assessing the toxicity of 2 mg/kg GRFT, administered systemically i.e., subcutaneously or intraperitoneally, to determine the potential adverse effects of GRFT in case of absorption. As shown in Figure 6A, the animals displayed similar body weights throughout the experiment, whether treated with GRFT or PBS, regardless of the administration route. The same observation was made for the relative spleen weights (Figure 6B). In accordance to these results, no activation of spleen B- or T-cells was found after GRFT administration regardless of the route, as evaluated using B220 and CD69 markers, respectively (Figure 6C,D). These results further demonstrated that GRFT has an excellent safety profile as a microbicide.

## 4. Discussion

### 4.1. GRFT’S Effects on mPBMCs Validate the Use of a Murine Model for Safety Studies

To validate the use of a murine model, we evaluated the toxicological effects of GRFT on PMBCs purified from mouse blood samples, in light of data described in our previous work on human PBMCs and cervico-vaginal cell lines [12]. As shown above, purified mPBMCs were not, or only moderately, affected by high concentrations of GRFT, in terms of viability and mitogenicity. In addition, IL-1b, IL-10 and TNF-α amounts in mPBMC culture supernatants were not altered by GRFT at concentrations of up to 4 μM. IL-6 concentrations were not affected by 1 μM GRFT, although a transient and moderate increase was observed after treatment with 4 μM at one day after treatment. These data indicate that GRFT does not boost release of the above four cytokines in mPBMCs, corroborating our previous observations that this lectin induces only minimal changes in the secretion of 27 cytokines and chemokines by human PBMCs, as assessed by multiplexed immunoassays [12]. Collectively, these findings suggested mouse to be a valid model for toxicological assessment of GRFT.

### 4.2. Even at Very High Doses, GRFT Does Not Cause Overt Toxicity in Mice

All mice survived single treatments with up to 50 mg/kg, and 14 daily treatments of 10 mg/kg GRFT. In addition, no GRFT treatment related adverse effects in behavior or fitness (body-weight gain or loss) were observed. These findings suggest that GRFT does not cause massive toxicity in mice after parenteral administration. Of note, the amounts of proteins used in these experiments were very high, since we aimed at recording the maximal effects that the lectin could trigger in adult mice. Recently, we demonstrated that GRFT is tolerated with minimal toxicity in juvenile guinea pigs after subcutaneous administration of 10 daily doses of 10 mg/kg [17]. Other lectins have also been studied in mice for their effects after parenteral administration. For instance, in an attempt to determine a well-tolerated dose in mice, Cyanovirin-N (CV-N) was injected beneath the skin of the upper back, and daily doses of 14 mg/kg caused weight loss in mice, while doses of 4.8 mg/kg and lower did not cause any signs of illness [20]. Our data revealed no GRFT treatment-related toxic effects on lungs, livers, kidneys, and hearts, as evaluated by organ weights and/or histology. However reversible splenomegaly was observed in mice treated with a single high dose (50 mg/kg) or chronically with 10 mg/kg GRFT. A study involving *Phaseolus vulgaris* lectin revealed that parenteral administration of the carbohydrate binding protein affects the extra-intestinal organs in suckling rats [21]. Indeed, it was shown that liver and spleen weights increased in animals treated parenterally with phytohemagglutinin and the authors suggested that the changes in organ weights are the result of an immunological cleansing/response process of these organs in response to the circulating PHA after the s.c. administration [21]. It is plausible that the observed splenomegaly in the case of GRFT reflects a nascent immune reaction from the animals as well. As shown above, B220-only positive splenocytes (CD19-) were elevated after 14 daily treatments with GRFT at 10 mg/kg, a difference that was not obtained after a week of recovery. Recently, a subset of murine splenic cells expressing B220 and lacking CD19 was described by Murakami and collaborators, and proposed to be involved in the formation of a follicular dendritic cell network [22], which plays a critical role in the immune reaction to foreign antigens [23]. It is unclear whether CD19^−/^B220^+^ cells increased after GRFT treatment have the same function in our model. In addition, CD69 expressing cells were slightly more abundant after chronic treatment with 10 mg/kg GRFT in this study; this effect was reduced after the recovery period. The effect of CD69 on spleen size remains controversial. While it was demonstrated that induction of the T-cell early activation marker CD69 in mice results in marked splenomegaly [24], CD69 knockout mouse spleens were shown to be enlarged compared with those of WT mice in tumor and collagen-induced arthritis challenge models [25,26].

### 4.3. GRFT Does Not Significantly Affect Blood Chemistry Markers of Toxicity

Subtle toxicity can be indirectly assessed by evaluating the effect of a drug on plasma concentrations of blood chemistry markers. A difference was observed in cholesterol levels, which were increased as a result of single 50 mg/kg or chronic 10 mg/kg doses of GRFT. However, the differences between the vehicle and GRFT groups were sharply reduced after recovery, as shown above. It has been suggested that splenomegaly could result from an increased uptake of unesterified Chol (and other circulating lipids) by splenic macrophages after a case study of a 31 year old man presenting a homogeneously enlarged spleen with an elevated serum total cholesterol [27]. Despite the observed increase in plasma Chol levels after GRFT administration, it is not clear whether high Chol levels are responsible for the described spleen enlargement or vice versa and there are insufficient data to correlate the two anomalies. After chronic treatment with 10 mg/kg GRFT, elevated ALP was observed, but the difference between control and treatment was reduced after recovery. Recently, we reported similar findings in GRFT treated guinea pigs [17]. Normal adult serum mainly contains liver-type ALP with small and variable amounts of intestinal and bone enzyme [28]. At this point, it is unclear whether the increased ALP levels observed reflected liver toxicity. Indeed, no changes were recorded for the other markers of liver toxicity assessed here, including Alb, TBILI, and ALP. Importantly, improvement of spleen enlargement, splenocyte activation, and Chol and ALP levels occurred only a few days post-treatment, indicating that even at high levels, GRFT does not cause irreparable damages to the spleen or the animal as a whole. These findings suggest that in a therapeutic setting, it might be more appropriate to administer the drug at longer time intervals, e.g., every other day, twice weekly, or weekly, rather than daily injections, if the dose of 10 mg/kg is to be maintained.

### 4.4. Chronic Intravaginal or Systemic Administration of 2 mg/kg GRFT Is Safe in Mice

It has been shown that intravaginal 2 mg/kg GRFT can inhibit HSV-2 [9]; a similar dose would be used for the mucosal prevention of HIV-1. As shown above, GRFT at this dose caused no detectable toxicity in mice after intravaginal treatment. Although it is unlikely for GRFT to be absorbed through mucosal surfaces after intravaginal administration in healthy subjects, it is possible for the drug to get into circulation in individuals suffering from ulcerative STDs. Indeed, it is widely accepted that STDs are an important risk factor for HIV and vice versa [29,30,31]. Therefore, we assessed the effects of 2 mg/kg GRFT delivered by intraperitoneal and subcutaneous administrations, respectively. No adverse effects were found in either case. These results confirm that GRFT has an excellent safety profile as a microbicide candidate, as previously shown using different systems [3,12,16,17]. Interestingly, GRFT was recently shown to significantly protect against genital herpes in a mouse model [9]. Meanwhile, a combination of GRFT and carrageenan displayed potent antiviral effects in mice infected with HSV-2 and human papillomavirus (HPV) [32]. These findings support the development of GRFT as a microbicide for the prevention of HIV/AIDS and other susceptible enveloped viruses.

## 5. Conclusions

In summary, our results indicate an excellent safety profile for GRFT in the murine model presented here, showing outstanding safety properties for this lectin, both in vivo and in vitro. Therefore, GRFT should be further developed as microbicide for the prevention of HIV/AIDS. In addition, this lectin could become a powerful weapon in the fight against blood-borne enveloped viruses in general, and should be considered for the treatment of AIDS as well, if administered via a parenteral route.

## Figures and Tables

**Table 1:** Effect of 14 daily s.c. doses of 10 mg/kg GRFT on blood chemistry parameters.

Parameter	Unit	Day 14		Day 21	
		PBS	GRFT	PBS	GRFT
Alb	g/dL	3.0 ± 0.4	3.0 ± 0.3	2.2 ± 0.4	2.9 ± 0.1
ALP	AU	100.0 ± 16.7	159.9 ± 31.0 ^1^	100.0 ± 14.0	144.5 ± 18.0 ^1^
ALT	AU	100.0 ± 36.7	86.5 ± 7.0	100.0 ± 73.0	57.1 ± 35.5
AMS	AU	100.0 ± 32.8	95.6 ± 7.5	100.0 ± 33.3	69.8 ± 6.2
BG	mg/dL	119.8 ± 24.7	124.8 ± 18.6	166.3 ± 28.3	138.0 ± 42.8
BUN	mg/dL	29.3 ± 3.3	25.0 ± 3.2	33.3 ± 6.7	26.0 ± 1.7
Ca	mg/dL	10.1 ± 0.4	10.9 ± 0.3	11.1 ± 1.2	10.6 ± 0.3
Chol	mg/dL	87.3 ± 5.4	118.8 ± 10.5 ^1^	69.0 ± 14.2	103.0 ± 3.6 ^1^
CREAT	mg/dL	0.4 ± 0.1	0.4 ± 0.0	0.2 ± 0.1	0.3 ± 0.1
GLOB	g/dL	3.1 ± 0.2	3.3 ± 0.1	4.1 ± 1.0	3.1 ± 0.1
P	mg/dL	10.4 ± 1.8	10.9 ± 0.9	10.3 ± 0.5	9.5 ± 0.9
TBILI	mg/dL	1.2 ± 0.5	1.2 ± 0.6	1.0 ± 0.5	0.5 ± 0.2
TP	g/dL	6.1 ± 0.6	6.3 ± 0.4	6.3 ± 0.6	5.9 ± 0.1

Mice were treated with GRFT at 10 mg/kg for 14 days and samples were analyzed at the end of treatment, as well as seven days post-treatment. AU, arbitrary units; Alb, albumin; ALP, alkaline phosphatase; ALT, alanine transaminase; AMS, amylase; BG, blood glucose; BUN, blood urea nitrogen; Ca, calcium; Chol, cholesterol; CREAT, creatinine; P, phosphorus; TBILI, total bilirubin; TP, total protein. ^1^ Statistical significance at *p* < 0.05.

**Table 2:** Effect of 14 daily s.c. doses of 10 mg/kg GRFT on a mouse hematological profile.

Cell Type	Parameter	Unit	Day 14		Day 21	
			PBS	GRFT	PBS	GRFT
**Leucocytes**	WBC	k/μL	7.9 ± 1.5	7.2 ± 0.5	8.3 ± 2.4	8.5 ± 1.6
	NE	k/μL	1.7 ± 0.6	2.7 ± 0.2	2.1 ± 1.3	2.9 ± 1.0
	LY	k/μL	5.4 ± 0.7	3.6 ± 0.4 ^1^	5.0 ± 0.6	4.3 ± 0.4
	MO	k/μL	0.6 ± 0.1	0.6 ± 0.1	0.9 ± 0.2	1.0 ± 0.5
	EO	k/μL	0.2 ± 0.1	0.3 ± 0.1	0.2 ± 0.3	0.3 ± 0.1
	BA	k/μL	0.1 ± 0.0	0.1 ± 0.0	0.1 ± 0.1	0.1 ± 0.0
**Erythrocytes**	RBC	M/µL	9.4 ± 0.5	9.1 ± 0.7	9.8 ± 1.1	10.1 ± 2.3
	Hb	g/dL	14.2 ± 1.0	13.2 ± 1.1	15.0 ± 0.8	15.1 ± 4.0
	HCT	%	57.9 ± 5.3	56.2 ± 5.5	59.4 ± 6.2	60.1 ± 14.9
	MCV	fL	61.4 ± 2.6	61.7 ± 1.2	60.6 ± 3.4	59.3 ± 1.5
	MCH	pg	15.1 ± 0.4	14.5 ± 0.6	15.3 ± 0.9	14.8 ± 0.7
	MCHC	g/dL	24.5 ± 0.6	23.5 ± 1.0	25.3 ± 1.7	25.0 ± 0.6
	RDW	%	17.3 ± 0.9	18.2 ± 0.5	18.0 ± 0.4	18.5 ± 0.4
**Thrombocytes**	PLT	k/μL	1571.3 ± 100.8	1397.0 ± 302.5	850.0 ± 43.5	949.0 ± 211.5
	MPV	fL	3.6 ± 0.1	3.7 ± 0.3	3.6 ± 0.2	3.8 ± 0.2

Mice were treated with GRFT at 10 mg/kg for 14 days and samples were analyzed at the end of treatment, as well as seven days post-treatment. WBC, white blood cell; NE, neutrophil; LY, lymphocyte; MO, monocyte; EO, eosinophil; BA, basophil; RBC, red blood cell; Hb, hemoglobin; HCT, hematocrit; MCV, mean corpuscular volume; MCH, mean cell hemoglobin; MCHC, mean cell hemoglobin concentration; RDW, red cell distribution width; PLT, platelet; MPV, mean platelet volume. ^1^ Statistical significance at *p* < 0.05.

## Supplementary Materials

The following are available online at www.mdpi.com/1999-4915/8/11/311/s1, Figure S1: Effect of GRFT on mPBMC death; Table S1: Effect of single subcutaneous dose of 50 mg/kg GRFT on blood chemistry parameters; Table S2: Effect of single subcutaneous dose of 50 mg/kg GRFT on mouse hematological profile.
