# Supplementary material for: Studies in a Murine Model Confirm the Safety of Griffithsin and Advocate Its Further Development as a Microbicide Targeting HIV-1 and Other Enveloped Viruses
Source: Viruses. 2016 Nov 17;8(11):311. doi: 10.3390/v8110311 (PMC5127025; doi:10.3390/v8110311)
Supplement: Supplementary file 1 [file viruses-08-00311-s001.pdf]

Supplementary Materials: Studies in a Murine Model Confirm the Safety of Griffithsin and Advocate its Further Development as a Microbicide Targeting HIV-1 and Other Enveloped Viruses

Joseph Calvin Kouokam, Amanda B. Lasnik and Kenneth E. Palmer

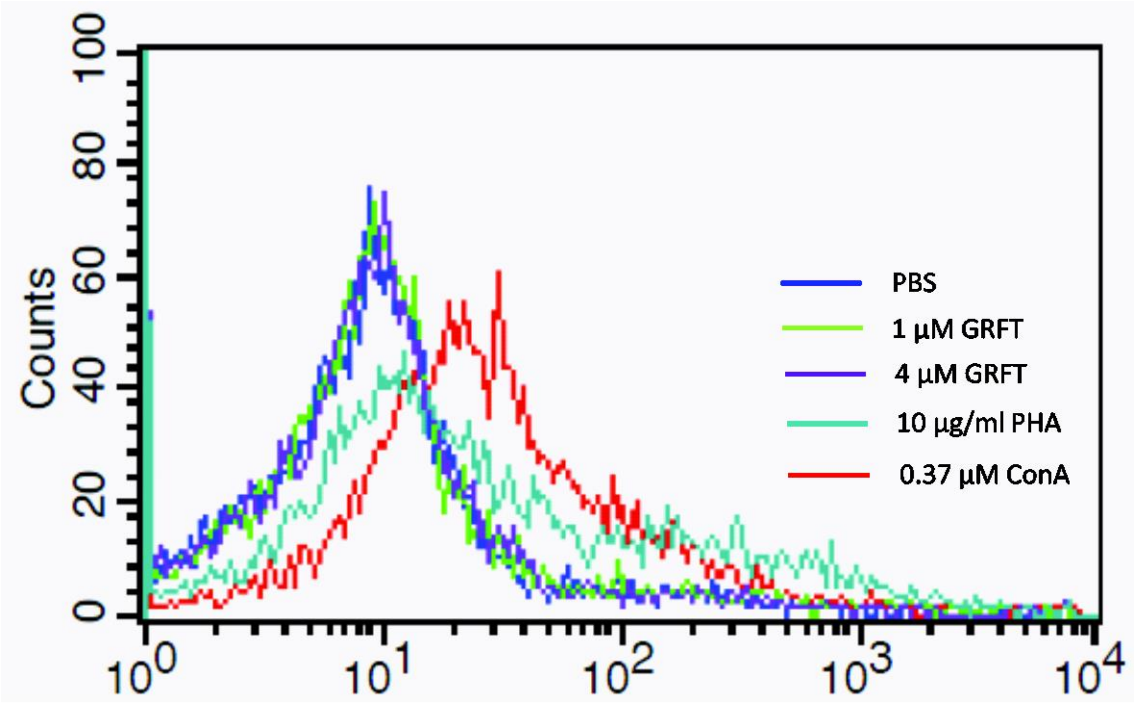

Figure S1. Effect of GRFT on mPBMC death. Cells were cultured for three days in presence of PBS, 1 and 4 µM GRFT, 0.37 µM Concanavalin A (ConA), and 10 µg/ml phytohemagglutinin (PHA), respectively. Prior to flow-cytometry analysis, cells were loaded with 1 µg/ml propidium iodide (PI). Flow cytometry histograms of mPBMCs treated with GRFT (1 or 4 µM) were similar to that obtained for PBS treated cells. PHA and ConA overtly induced cell death as reflected by a shift in histograms (increased PI staining) in comparison with PBS or GRFT treated cells.

Table S1. Effect of single subcutaneous dose of 50 mg/kg GRFT on blood chemistry parameters.

Parameter	Unit	7 Days Post Treatment		14 Days Post Treatment	
		PBS	GRFT	PBS	GRFT
ALB	g/dL	4.3 ± 0.4	3.8 ± 0.1	2.5 ± 0.5	3.0 ± 1.5
ALP	AU	100.0 ± 33.1	147.3 ± 7.8 [1]	100.0 ± 5.3	144.2 ± 49.3
ALT	AU	100.0 ± 64.1	76.4 ± 25.1	100.0 ± 13.1	159.0 ± 50.1
AMY	AU	100.0 ± 13.4	102.8 ± 3.9	100.0 ± 14.0	169.6 ± 76.9
TBIL	mg/dL	0.2 ± 0.0	0.5 ± 0.6	0.6 ± 0.3	0.6 ± 0.3
BUN	mg/dL	21.3 ± 4.2	21.7 ± 4.2	26.0 ± 1.0	27.3 ± 7.6
CA	mg/dL	11.3 ± 0.1	11.6 ± 0.4	11.0 ± 0.8	14.0 ± 4.1
PHOS	mg/dL	10.5 ± 0.4	11.2 ± 1.4	10.6 ± 0.9	13.8 ± 4.4
CRE	mg/dL	<0.2	<0.2	0.3 ± 0.1	0.3 ± 0.0
GLU	mg/dL	186.7 ± 41.3	197.0 ± 39.6	171.7 ± 34.8	261.8 ± 47.7
Na^+	mEq/L	158.7 ± 0.6	160.0 ± 2.6	n.d.	n.d.
K^+	mEq/L	>8.5	>8.5	n.d.	n.d.
TP	g/dL	5.8 ± 0.3	5.6 ± 0.4	6.1 ± 0.4	7.5 ± 2.5
GLOB	g/dL	1.5 ± 0.1	1.7 ± 0.4	3.6 ± 0.5	4.4 ± 1.3
Cholesterol	mg/dL			63.3 ± 9.1	107.0 ± 48.6

ALB, albumin; ALP, alkaline phosphatase; ALT, alanine transaminase; AMY, amylase; BUN, blood urea nitrogen; Ca, calcium; CHOL, cholesterol; CRE, creatinine; GLOB, globulin; GLU, glucose; PHOS, phosphorus; TBIL, total bilirubin; TP, total protein; AU, arbitrary units; n.d., not determined.
[1] Statistical significance at $p < 0.05$.

Table S2. Effect of single subcutaneous dose of 50 mg/kg GRFT on mouse hematological profile.

Cell Type	Parameter	Unit	PBS	GRFT
Leucocytes	WBC	k/μL	7.9 ± 1.2	9.0 ± 3.2
	NE	k/μL	2.1 ± 0.3	2.0 ± 0.7
	LY	k/μL	4.9 ± 0.7	5.9 ± 2.3
	MO	k/μL	0.7 ± 0.2	0.9 ± 0.2
	EO	k/μL	0.1 ± 0.1	0.1 ± 0.0
	BA	k/μL	0.0 ± 0.0	0.1 ± 0.0
Erythrocytes	RBC	M/μL	10.0 ± 1.9	10.5 ± 0.7
	Hb	g/dL	14.5 ± 2.5	14.9 ± 0.6
	HCT	%	61.5 ± 11.8	64.6 ± 5.0
	MCV	fL	61.4 ± 0.8	61.8 ± 1.0
	MCH	pg	14.5 ± 0.6	14.3 ± 0.7
	MCHC	g/dL	23.7 ± 0.8	23.2 ± 1.4
	RDW	%	17.5 ± 0.7	17.4 ± 0.1
Thrombocytes	PLT	k/μL	1294.7 ± 213.9	1222.5 ± 177.4
	MPV	fL	3.3 ± 0.3	3.3 ± 0.3

Mice were treated with a single dose of 50 mg/kg GRFT and sacrificed at 14 days for analysis. WBC, white blood cell; NE, neutrophil; LY, lymphocyte; MO, monocyte; EO, eosinophil; BA, basophil; RBC, red blood cell; Hb, hemoglobin; HCT, hematocrit; MCV, mean corpuscular volume; MCH, mean cell hemoglobin; MCHC, mean cell hemoglobin concentration; RDW, red cell distribution width; PLT, platelet; MPV, mean platelet volume. All values were similar between both groups ($p > 0.05$).

© 2016 by the authors. Submitted for possible open access publication under the terms and conditions of the Creative Commons Attribution (CC-BY) license (http://creativecommons.org/licenses/by/4.0/).
